# Ausbildung in COVID-19-Pandemie-Zeiten: Wie bewerten Medizinstudierende einen interaktiven, videobasierten Distanzunterricht am Patienten im Fach Hals-Nasen-Ohren-Heilkunde?

**DOI:** 10.1007/s00106-021-01117-x

**Published:** 2021-11-23

**Authors:** Ingmar Seiwerth, S. Bartel, M. Herzog, G. Schumann, M. K. Pein, A. Gey, S. K. Plontke

**Affiliations:** 1 Universitätsklinik und Poliklinik für Hals-Nasen-Ohren-Heilkunde, Kopf- und Hals-Chirurgie, Martin-Luther-Universität Halle-Wittenberg, Universitätsklinikum Halle (Saale), Ernst-Grube-Str. 40, 06120 Halle (Saale), Deutschland; 2 Klinik für HNO-Krankheiten, Kopf- und Halschirurgie, Carl-Thiem-Klinikum Cottbus, Cottbus, Deutschland; 3 Zentraler Dienst 1 – Information und Kommunikation, Universitätsklinikum Halle (Saale), Halle (Saale), Deutschland

**Keywords:** Unterricht am Patienten, Digitalisierung, Digitale Lehre, Medizinische Lehre, Kontaktbeschränkungen, Teleteaching, Bedside teaching, Digitalization, Digital education, Medical education, Contact restrictions, Teleteaching

## Abstract

**Hintergrund:**

Der Beginn der ersten SARS-CoV-2-Pandemie-Welle im März 2020 erforderte erhebliche Umstellungsmaßnahmen in der medizinischen Lehre mit vollständigem Verzicht auf direkten Kontakt von Studierenden und Patienten. Vor diesem Hintergrund wurde das Lehrformat „Unterricht am Patienten“ (UaP) als interaktiver, videobasierter Distanzunterricht am Patienten etabliert und durchgeführt.

**Fragestellung:**

Ziel der Studie war die Erfassung der studentischen Beurteilung dieses Lehrkonzeptes im Fach Hals-Nasen-Ohren-Heilkunde.

**Material und Methoden:**

Die Live-Übertragung erfolgte aus einem HNO-Untersuchungsraum zu den im Hörsaal befindlichen Studierenden, welche mit den Patienten interagieren konnten. Makro-, mikro- und endoskopische Untersuchungsbefunde wurden in Echtzeit in den Hörsaal übertragen. Die Evaluation erfolgte anhand eines Online-Fragebogens, welcher 13 geschlossene Fragen (Likert-Skala) beinhaltete, sowie als offenes Feedback in freier Textform.

**Ergebnisse:**

Die Rücklaufquote lag bei 16,8 % (42 von 250 Studierenden). Davon hatten 85,7 % einen positiven Gesamteindruck, und Tenor war, dass das Konzept angesichts der Umstände gut umgesetzt wurde. Dennoch könne auf einen direkten Patientenkontakt eher nicht verzichtet werden, auch wenn eine teilweise Kompensation durch Videoschaltung möglich sei. Insgesamt wurde das Lehrkonzept als lehrreich empfunden, und die Studierenden konnten sich vorstellen, zukünftig häufiger auch ein solches UaP-Lehrkonzept zu nutzen.

**Schlussfolgerung:**

Dieses Lehrkonzept kann den direkten Patientenkontakt nicht ersetzen, stellt jedoch speziell im HNO-Gebiet eine gute Alternative dar, wenn durch pandemiebedingte Umstände ein „klassischer“ UaP nicht möglich ist. Aspekte des interaktiven, videobasierten Distanzunterrichts am Patienten könnten auch zukünftig in andere Lehrformate integriert werden.

## Abstract (en)

**Background:**

The first wave of the SARS-CoV‑2 pandemic required substantial changes in the teaching of medical students, with strict avoidance of direct contact between students and patients. Therefore, the teaching format “bedside teaching” was implemented and conducted as an interactive video-based distance bedside teaching.

**Objective:**

The objective of this study was to analyze a students’ evaluation of this teaching concept in otorhinolaryngology.

**Materials and methods:**

From an ENT examination room, the situation was transmitted live to the students in a lecture hall, who could interact with the patients through a video connection. Macro-, micro-, and endoscopic images were transmitted into the lecture hall in real time. Evaluation was performed by means of an online questionnaire with 13 questions (Likert scale) as well as by free-text feedback.

**Results:**

The response rate was 16.8% (42 of 250 students). Overall, 85.7% had a positive impression, and it was generally considered that the concept was well implemented in light of the special situation. However, students would rather not renounce direct patient contact, even if a certain compensation by video transmission was reported. Overall, this teaching concept was considered as educative, and students could imagine using such a teaching concept more often in the future.

**Conclusion:**

This teaching model cannot replace classical bedside teaching, but represents a good alternative—particularly in otorhinolaryngology—if classical bedside teaching is not possible due to the pandemic situation. Aspects of the interactive video-based distance bedside teaching could be implemented into classical teaching concepts in the future.

## Hintergrund und Fragestellung

Der Beginn der ersten SARS-CoV-2-Pandemie-Welle in Deutschland im März 2020 lag nur wenige Wochen vor dem Beginn des Sommersemesters (SS) an den medizinischen Fakultäten in Deutschland. Die staatlichen Verordnungen zur Eindämmung umfassten auch strenge Kontaktbeschränkungen und erforderten erhebliche Umstellungsmaßnahmen in der Lehre an den Universitäten im Allgemeinen und speziell in der Ausbildung von Studierenden im Fach Humanmedizin einschließlich des vollständigen Verzichts auf Veranstaltungen mit direktem Kontakt von Studierenden und Patientinnen und Patienten. Dies führte zur Einführung – oder, wo schon zum Teil genutzt, zur deutlich vermehrten Nutzung – virtueller oder online-basierter Lehrveranstaltungen, wie z. B. Vorlesungsaufzeichnungen, Seminare, Unterrichte am Krankenbett (UaP), Prüfungen, Materialsammlungen und Tutorials mit und ohne Nutzung von Lernplattformen oder Gruppen- und Individualbetreuung via E‑Mails und Videokonferenzen [1, 5, 13, 15]. Stöver et al. [15] konnten aus einer Befragung der 39 Direktorinnen und Direktoren deutscher Universitäts-HNO-Kliniken ermitteln, dass bei 97,4 % infolge der SARS-CoV-2-Pandemie solche Lehrformate eingesetzt und Präsenzprüfungen von ehemals 89,7 auf 53,8 % der Universitätskliniken reduziert wurden. Offergeld et al. beschrieben jedoch mangelnde Ressourcen zur Einführung solcher Lehrformate und gaben eine kritische Einschätzung gegenüber der aktuellen virtuellen oder online-basierten Lehrsituation [10].

Im Sommersemester konzentrieren sich jährlich an unserer Fakultät die curricularen und nichtcurricularen Lehraktivitäten im Fach Hals-Nasen-Ohren-Heilkunde. Bei der kurzfristigen Umgestaltung der medizinischen Ausbildung als Reaktion auf die Pandemiebedingungen war es ein erklärtes Ziel, dass die Studierenden kein Semester „verlieren“, d. h. dass die Herausforderungen in der Lehre nicht zu einer Verlängerung der Studiendauer führen. Dabei stellte der curriculare Unterricht am Patienten (UaP) unter gleichzeitigem Verbot eines direkten Patientenkontakts eine besondere Problematik dar. Wir haben dies durch eine ärztlich moderierte und studentisch geführte, aber ärztlich vermittelte Distanzanamnese und -Untersuchung mittels Live-Übertragung aus einem HNO-Untersuchungszimmer in den Hörsaal unter adäquaten Hygienebedingungen realisiert. Die Distanzuntersuchung beinhaltete neben äußerlichen, makroskopischen Aspekten auch endoskopische und mikroskopische Befunde. Nach Ende der Veranstaltungsreihe wurden die Studierenden um eine Einschätzung dieser Lehrveranstaltung mithilfe eines Fragebogens gebeten.

Ziel der Untersuchung war die Erfassung der studentischen Beurteilung eines „interaktiven, videobasierten Distanzunterrichts am Patienten“ im Fach Hals-Nasen-Ohren-Heilkunde.

## Methode

### Zeitlicher Ablauf

Ursprünglich war der Beginn des „klassischen“ UaP am 06.04.2020 geplant. Am 13.03.2020 wurde bekannt, dass der universitäre Lehrbetrieb pandemiebedingt eingestellt werde. Am 07.04.2020 wurde festgelegt, dass der digitale universitäre Lehrbetrieb am 20.04.2020 beginnen sollte. Präsenzpflichtige Veranstaltungen sollten entsprechend der aktuell gültigen Eindämmungsverordnung angepasst werden. Der Unterricht erfolgte in Form eines „interaktiven, videobasierten Distanz-UaP“ im Zeitraum vom 18.05.2020–09.07.2020.

### Durchführung

**Figure 1:**
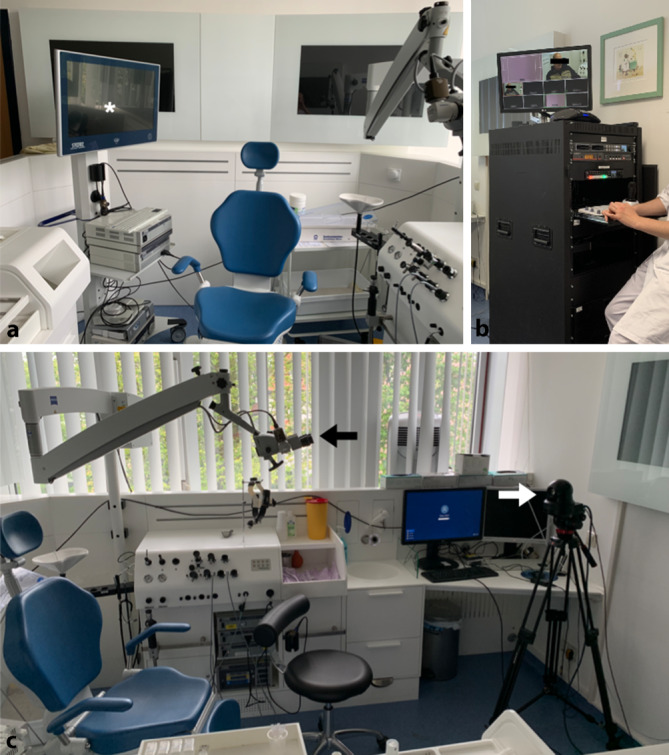

Die Patientin oder der Patient wurde gebeten, im Untersuchungsraum auf dem Untersuchungsstuhl (Abb. 1) Platz zu nehmen, gegenüber einer für die Untersuchung und Gesprächsführung verantwortlichen ärztlichen Lehrkraft. Eine weitere, ebenfalls im Untersuchungsraum befindliche ärztliche Lehrkraft überwachte und koordinierte die technische Übertragung der Bild- und Tonsignale sowie die Kommunikation mit dem im Hörsaal befindlichen Moderator. Die Studierenden saßen in einer Gruppengröße von jeweils 30 Personen unter strenger Berücksichtigung der Hygienevorgaben nach der zum aktuellen Zeitpunkt geltenden Pandemie-Eindämmungsverordnung [7] (Abstand, Maske, Händedesinfektion) in einem Hörsaal mit circa 150 Plätzen.

Die Anamnese wurde durch die Studierenden erhoben. Die Studierenden formulierten die Fragen an den Moderator im Hörsaal, welcher die Fragen in den Untersuchungsraum weitergab, sodass diese direkt von der Patientin oder vom Patienten beantwortet werden konnten. Die Untersuchung erfolgte dann durch die ärztliche Lehrkraft im Untersuchungsraum auf „Anweisung“ durch die Studierenden im Hörsaal. Die Durchführung der Untersuchung einschließlich der entsprechenden Befunde wurden jeweils mittels einer Makrokamera (Saalkamera), eines Mikroskops und/oder eines Endoskops jeweils mit Kamera erfasst und in den Hörsaal mittels Videoprojektor auf eine Leinwand übertragen. Zusätzliche Befunde wie beispielsweise Laborparameter, audiologische und neurootologische Daten oder radiologische Befunde wurden im Bedarfsfall durch den Moderator im Hörsaal präsentiert.

Für eine Distanz-UaP-Einheit war eine Dauer von 2 h und 15 min vorgegeben. Jede Distanz-UaP-Einheit folgte im Ablauf einem entsprechenden „Drehbuch“, welches im Voraus erstellt wurde. Ergänzend erfolgte jeweils eine weiterführende theoretische Erläuterung des Krankheitsbildes durch den Moderator im Hörsaal mit Seminarcharakter. Jede Distanz-UaP-Einheit war thematisch einem HNO-Untergebiet zugeordnet (UaP1: Untersuchungstechniken + UaP2: Ohr (Kalenderwoche [KW] 21 und 25), UaP3: Nase (KW 22 und 26), UaP4: Mundhöhle/Hals/Pharynx (KW 23 und 27), UaP5: Larynx (KW 24 und 29)). Organisatorisch wurde pro KW (Montag jeweils drei und Donnerstag eine UaP-Einheit) ein UaP-Themengebiet, wenn möglich am gleichen Patienten, absolviert. Die als Moderator wirkende ärztliche Lehrkraft war in der entsprechenden KW für das jeweilige Thema eingeteilt, dann erfolgte ein Wechsel. Der Untersuchungsraum wurde während der gesamten Unterrichtsperiode von einem Kernteam von drei ärztlichen Lehrkräften bedient, die sich wöchentlich so abwechselten, dass der Untersuchungsraum pro UaP-Einheit mindestens zu zweit ärztlich besetzt war.

Was den Personalaufwand betrifft, so waren je UaP-Einheit insgesamt fünf bis sechs Personen erforderlich, davon mindestens drei ärztliche und zwei wissenschaftliche oder technische Mitarbeiter. Beim ärztlichen Personal wurde angesichts der fehlenden Erfahrung mit dem neuen Konzept darauf geachtet, nur erfahrene Lehrkräfte einzusetzen. So war der Moderator entweder ein Facharzt, Oberarzt oder Chefarzt, während im Untersuchungsraum ärztliche Lehrkräfte in weit fortgeschrittenem Weiterbildungsstadium oder mit Facharztstatus eingesetzt wurden. Im Vergleich hierzu waren ursprünglich in der Planung für die Durchführung des „klassischen“ UaP, welche vor Pandemiebeginn feststand, fünf bis sechs ärztliche Mitarbeiter (Weiterbildungsassistenten, teilw. auch Fachärzte und Oberärzte) pro UaP-Einheit vorgesehen.

Da eine UaP-Einheit mehrmals pro Woche bei unterschiedlichen Gruppen durchgeführt wurde, konnten nicht immer gewährleistet werden, dass die Patientinnen und Patienten bei allen Gruppen die gleichen waren. Alle Studierenden mussten im Verlauf des Sommersemesters an insgesamt vier Distanz-UaP-Unterrichtseinheiten teilnehmen. Ab UaP2 hatten die Studierenden bis zur nächsten UaP-Einheit (i. d. R. eine Woche) Zeit, sich auf das nächste Thema vorzubereiten. Dabei sollten auch vorgegebene wissenschaftliche Publikationen zum jeweiligen Thema bewertet werden.

### Technische Umsetzung

(Abb. 1).

Die Konzeption der Übertragung, die Zusammenstellung der technischen Geräte und die Montage des Übertragungsturms im Untersuchungsraum erfolgte durch Mitarbeiter des Zentralen Dienstes ZD 1, Information und Kommunikation des Universitätsklinikums Halle.

Als Bildquellen dienten ein Storz-AIDA-System (Fa. Karl Storz SE & Co. KG, Tuttlingen) mit Kamerakopf und eine auf einem Stativ (Manfrotto 546B + MVH502A „Fluid Video Head“, Fa. Vitec Imaging Solutions Spa, Cassola, Italien) montierte Kamera (SONY EVI-H100V Full HD, „pan, tilt, zoom“ [PTZ], 20 × optisches Zoom, Fa. Sony Group Corporation, Tokio, Japan) mit PTZ-Steuerung (SONY RM-BR300 PTZ Steuerung, Fa. Sony Group Corporation, Tokio, Japan). Die zwei Bildquellen wurden über Signalwandler (Blackmagic Mini Converter UpDownCross HD, Fa. Blackmagic Design Pty. Ltd, Port Melbourne, Australien) an einen High Definition Multimedia Interface (HDMI)/Serial Digital Interface (SDI)-Bildmischer (Blackmagic ATEM Television Studio HD, Fa. Blackmagic Design Pty. Ltd, Port Melbourne, Australien) angeschlossen, um ein einheitliches Bildformat 1080p zu gewährleisten. Mit dem Bildmischer bestand die Möglichkeit, ohne Unterbrechung von Bildquelle zu Bildquelle umzuschalten oder auch Bild in Bild darzustellen. Zur Tonübertragung diente ein Headset-Mikrofon (Sennheiser EW-100 G3 + Bodypack, Fa. Sennheiser electronic GmbH & Co. KG, Wedemark), welches an den Bildmischer angeschlossen und im Ausgang dem Stream beigemischt wurde. Die Übertragung des Streams über das Haus-IT-Netzwerk erfolgte mit einem Haivision Makito2 Encoder/Decoder (Fa. Haivision, Montreal, Kanada), welcher an den Ausgang des Bildmischers angeschlossen wurde. Somit konnte der SRT-Videostream an einer anderen Stelle des Haus-IT-Netzwerks mit dem Haivision Makito2 Decoder entnommen werden, welcher dann an einen Bildschirm oder Videoprojektor angeschlossen wurde. Zur Gewährleistung des Tonrückkanals vom Hörsaal in den Untersuchungsraum wurde ein mobiles „Digital Enhanced Cordless Telecommunications“(DECT)-Telefon (mit externem Mikrofon), welches sich beim Moderator befand, und ein DECT-Konferenztelefon im Untersuchungsraum benutzt. Somit war eine direkte Verständigung möglich.

Die Empfangseinheit im Hörsaal wurde durch mindestens eine weitere Person (je nach Verfügung technisches und/oder ärztliches bzw. wissenschaftliches Personal) überwacht.

### Evaluation

Die Beurteilung des Distanz-UaP erfolgte Anhand eines Online-Fragebogens, zu dessen freiwilliger Beantwortung die Studierenden vom Studiendekanat am 21.01.2021 per E‑Mail eingeladen wurden. Eine Erinnerungs-E-Mail wurde am 25.02.2021 verschickt. Die Erstellung und Auswertung erfolgte mittels der Software EvaSys (Fa. evasys GmbH, Lüneburg). Die Beantwortung der Fragen erfolgte anonymisiert. Der Fragebogen bestand, neben Angaben zu Geschlecht und Alter, aus 13 geschlossenen Fragen, welche im Sinne einer 5‑stelligen Likert-Skala formuliert waren. Zudem bestand die Möglichkeit, eine freie Antwort für Feedback (Wünsche, Anregungen und Kritik) zu formulieren. Die offenen Antworten wurden kategorisiert im Sinne einer thematischen Zusammenfassung ähnlicher Aussagen und nach Häufigkeit ausgewertet.

## Ergebnisse

### Allgemein

Im Sommersemester 2020 nahmen 250 Studierende an der Lehrveranstaltung UaP teil. Die Anzahl der erfassten Fragebögen betrug *n* = 42, was einer Rücklaufquote von 16,8 % entsprach. Etwa die Hälfte der Teilnehmenden (52,4 %) waren weiblich, 45,2 % männlich und 2,4 % divers; 42,9 % der Teilnehmer waren zwischen 21 und 23 Jahre alt, während die zweithäufigste Altersgruppe mit 31 % die Gruppe der 24- bis 26-Jährigen darstellte.

### Geschlossene Fragen

**Figure 2:**
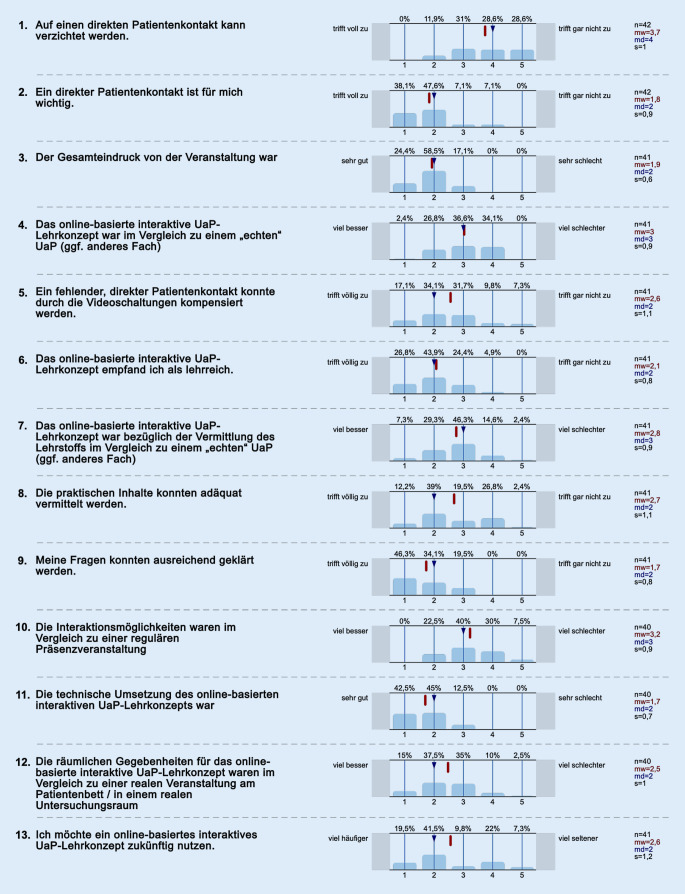

Die ausführliche Auswertung der einzelnen Fragen ist in Abb. 2 dargestellt.

Für mehr als die Hälfte (57,2 %) der an der Befragung teilnehmenden Studierenden konnte auf direkten Patientenkontakt nicht verzichtet werden (Frage 1). Für 85,7 % war ein direkter Patientenkontakt wichtig (Frage 2). Einen positiven Gesamteindruck von der Veranstaltung hatten 82,9 % der Teilnehmenden (Frage 3). Das interaktive Distanz-UaP-Lehrkonzept wurde im Vergleich zu einem „echten“ UaP insgesamt weder besser noch schlechter eingeschätzt (Frage 4). Dass ein fehlender, direkter Patientenkontakt durch die Videoschaltungen kompensiert werden konnte, traf für 51,2 % zu (Frage 5), und 70,7 % empfanden das interaktive Distanz-UaP-Lehrkonzept als lehrreich (Frage 6). Dieses Format war bezüglich der Vermittlung des Lehrstoffs im Vergleich zu einem „echten“ UaP für 46,3 % im Wesentlichen gleichwertig (Frage 7), während die praktischen Inhalte für 51,2 % adäquat vermittelt (Frage 8) und für die überwältigende Mehrheit (80,4) die persönlichen Fragen ausreichend geklärt werden konnten (Frage 9). Für 40 % der an der Befragung Teilnehmenden waren die Interaktionsmöglichkeiten im Vergleich zu einer regulären Präsenzveranstaltung weder besser noch schlechter, während 37,5 % bei dieser Frage zu „schlechter“ neigten und 22,5 % der Studierenden die Interaktionsmöglichkeiten sogar als besser beurteilten (Frage 10).

Die technische Umsetzung des interaktiven Distanz-UaP-Lehrkonzepts wurde mehrheitlich als gut oder sehr gut (87,5 %) empfunden (Frage 11), und die räumlichen Gegebenheiten für das interaktive Distanz-UaP-Lehrkonzept wurden im Vergleich zu einer Veranstaltung mit direktem Patientenkontakt und persönlicher Anwesenheit im Untersuchungsraum von 52,5 % als besser und von 35 % als gleichwertig (35 %) angegeben (Frage 12). Ein interaktives Telemedizin-Lehrkonzept im UaP möchten zukünftig 61 % der Befragten nutzen (Frage 13).

### Offene Antworten

(Abb. 3).

**Figure 3:**
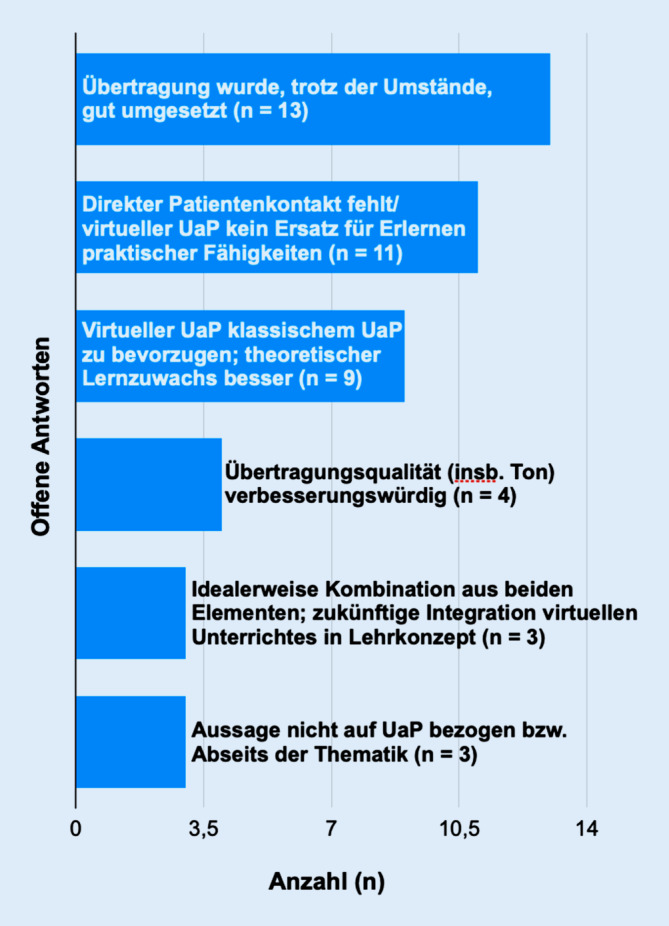

Es wurden 17 offene Feedbacks mit insgesamt 43 Aussagen formuliert. Die am häufigsten vorkommende Kernaussage war, dass das Konzept des Distanz-UaP trotz der erschwerten Umstände vom Lehrteam gut umgesetzt wurde (*n* = 13). Beispielsweise wurde mehrfach die Darstellung der Befunde auf der Leinwand als positiv und lehrreich aufgefasst.

Gleichzeitig wurde jedoch auch angemerkt, dass ein direkter Patientenkontakt fehlte bzw. das Distanz-UaP-Konzept kein Ersatz für das Erlernen praktischer Fähigkeiten sei (*n* = 11).

Am dritthäufigsten (*n* = 9) wurde die Aussage formuliert, dass ein Distanz-UaP dem „klassischen“ UaP zu bevorzugen sei. So sei der „theoretische Lernzuwachs“ mit diesem Format besser. Insbesondere seien die räumlichen Bedingungen im Unterschied zur Situation im engen Untersuchungsraum vorteilhaft, da die Studierenden die Befunde besser sehen und gleichzeitig mitschreiben konnten. Auch wurde die Betreuung durch die Tutoren beim früheren „klassischen“ UaP als unzureichend beschrieben, da diese nebenbei oft klinischen Verpflichtungen in der Patientenbetreuung nachkommen mussten.

Die Übertragungsqualität bemängelten vier Teilnehmende, insbesondere den Ton, und stuften diese als verbesserungswürdig ein. In drei Fällen wurde idealerweise eine Kombination aus beiden Elementen bzw. eine zukünftige Integration dieser Art des Unterrichts in das Lehrkonzept empfohlen.

## Diskussion

Insgesamt hatten 85,7 % der an der Befragung Teilnehmenden einen positiven Gesamteindruck von der Unterrichtsveranstaltung, und der Tenor war, dass das Konzept angesichts der Umstände gut umgesetzt wurde.

Dennoch könne auf einen direkten Patientenkontakt eher nicht verzichtet werden, auch wenn in gewissem Maße eine Kompensation durch Videoschaltung möglich sei. Nachteilig wurde auch die schlechtere Interaktionsmöglichkeit beschrieben.

Es kristallisierte sich sowohl in den geschlossenen Fragen als auch den offenen Antworten heraus, dass das Lehrkonzept als lehrreich empfunden wurde und die Studierenden sich vorstellen konnten, zukünftig häufiger auch ein solches UaP-Lehrkonzept zu nutzen.

In einem ähnlichen Format adaptierten Haucke et al. [3] das Lehrmodul „Interprofessionelles Telekonsil“ für die Lehre im Blockpraktikum „Innere Medizin“: Dabei begleiteten jeweils 20 im Hörsaal befindliche Studierende unter Einhaltung der geltenden Hygienevorschriften (Abstand, Mund-Nasen-Schutz, Händedesinfektion) eine Ärztin bzw. einen Arzt per ferngesteuertem Telepräsenzsystem (Double2, Fa. Double Robotics, Burlingame, CA, USA) über die Station und führten Anamnesegespräche, welche von einer zweiten ärztlichen Lehrkraft im Hörsaal moderiert wurden. Zur Evaluation dienten drei Fragen, in welchen der didaktische Aufbau (MW: 2,1; SD 0,9), der organisatorische Ablauf (MW 1,8; SD 0,7) und die Vermittlung der Lerninhalte (MW 1,6; SD 0,7) auf einer 5‑stufigen Likert-Skala von „trifft völlig zu“ (1) bis „trifft gar nicht zu“ (5) jeweils mit guten Ergebnissen bewertet wurden. Bei Hofmann et al. [4] wurden traditionelle UaP-Einheiten ebenfalls pandemiebedingt adaptiert: Eine ärztliche Lehrkraft moderierte eine zoombasierte (Fa. Zoom Video Communications Inc., San Jose/CA, USA) Videokonferenz für Studierende bei an COVID-19 erkrankten Patienten am Patientenbett. In einem Likert-Skala-basierten Fragebogen, welcher im Anschluss von 14 Studierenden beantwortet wurde, wurde dieses Konzept sehr positiv aufgefasst und als lehrreich empfunden.

Während in diesen Studien im Wesentlichen die Anamnese und die Gesprächsführung vordergründig waren, muss bei der Bewertung unserer Arbeit berücksichtigt werden, dass HNO-spezifische Untersuchungstechniken zusätzlich zur Anamnese und Gesprächsführung einen wichtigen Bestandteil des HNO-UaP darstellen. Dies kann als limitierender Faktor dieser Studie diskutiert werden, da bei Fragen, in denen ein Vergleich des Distanz-UaP mit einem „klassischen“ UaP erforderlich ist, dieser mit UaP aus anderen Fachrichtungen (welche die Studierenden i. d. R. in den Semestern vor der Pandemie in klassischer Form absolviert hatten), in denen der praktische Untersuchungsteil möglicherweise weniger aufwendig ist, verglichen wird. Im HNO-Fachgebiet erfordert eine gründliche Befunderhebung ein gewisses Maß an praktischen Fertigkeiten, deren korrekte Durchführung sehr erfahrungsabhängig ist, beispielweise bei der Ohrmikroskopie oder der Erhebung von Pharynx- und Larynxbefunden. Im „klassischen“ Präsenz-HNO-UaP ist es eines der Lernziele, den Studierenden diese Untersuchungstechniken beizubringen. Im interaktiven, videobasierten Distanz-UaP werden diese Techniken den Studierenden erläutert und demonstriert und somit nur theoretisch mit visueller Demonstration vermittelt. Auf die praktische Umsetzung und Überprüfung muss verzichtet werden, womit ein wichtiger Aspekt bei der Vermittlung von Fertigkeiten, z. B. gemäß der 4‑Schritt-Methode nach Peyton [9, 12], wegfällt.

Demgegenüber bietet sich im videobasierten Distanz-UaP den Studierenden die Möglichkeit, durch die Bildübertragung der jeweiligen Untersuchung durch die ärztliche Lehrkraft makro-, mikro- und endoskopische Befunde demonstriert zu bekommen, welche die Studierenden in dieser Form aufgrund der fehlenden Erfahrung bei der Untersuchungstechnik in einem klassischen UaP sehr wahrscheinlich nicht selbst in dem gleichen Maße hätten erheben können. Zudem ist im videobasierten Distanzunterricht eine gewisse Konstanz und Qualität der Lehre durch den Einsatz von sehr erfahrenen ärztlichen Lehrkräften als Moderatoren zu erwarten, was beim klassischen UaP nicht immer gewährleistet werden kann.

Mit Einschränkung der Präsenzlehre musste die Umstellung auf neue Lehrformate innerhalb kürzester Zeit erfolgen, was weltweit die medizinische Lehre vor deutliche Herausforderungen stellte. In der Arbeit von Stöver et al. (2021) wurden auch die technische Qualifikation der Mitarbeiter sowie eine fehlende methodisch-didaktische Kompetenz als problematisch beschrieben [15]. In unserem Fall konnten wir, in enger Abstimmung mit der medizinischen Fakultät, auf eine Übertragungstechnik zurückgreifen, welche aus hauseigenen Mitteln vom „Zentralen Dienst Information und Kommunikation des Universitätsklinikums“ (ZD 1) zum Zweck der Bild- und Tonübertragung aus dem Operationssaal in den Hörsaal aus einzelnen, kommerziell erhältlichen Komponenten zusammengestellt wurde und in den vergangenen Jahren bei den „Halleschen OP-Wochen“, einer außercurricularen, interdisziplinären, interprofessionellen, jährlich stattfindenden Veranstaltung mit Live-Übertragung aus dem OP [2], wie auch bei Fortbildungsveranstaltungen, beispielsweise bei Operationskursen für Mikrochirurgie des Ohrs [16], zum Einsatz kam. Es bestand somit schon Erfahrung im Umgang mit diesem Übertragungssystem. Der technische Support wurde während der Übertragung gewährleistet, sodass sich die ärztlichen Lehrkräfte im Wesentlichen auf die Didaktik konzentrieren konnten. Allerdings wären zur Optimierung der teilweise als verbesserungswürdig eingestuften Tonqualität sowie zur Bereithaltung eines Backup-Systems im Fall eines technischen Defekts weitere finanzielle Mittel erforderlich. In einer bundesweiten Umfrage zu digitalen Rahmenbedingungen der curricularen Lehre an Universitätskliniken und akademischen Lehrkrankenhäusern beschrieben Offergeld et al. (2021) offensichtliche Diskrepanzen zwischen verfügbaren Ressourcen und digitalisierten Lehrinhalten. Auch die Kommunikation mit der Medizinischen Fakultät, die digitale Infrastruktur und insbesondere oftmals mangelnde Kollaboration mit den zentralen Supportstrukturen wurden oft als problematisch beschrieben [10].

Was das Ausmaß des personellen, zeitlichen und technischen Aufwands für die Klinikorganisation im Vergleich zu einem „klassischen“ UaP betrifft, so ist hier ein direkter Vergleich schwierig. Auch wenn beim videobasierten Distanz-UaP je UaP-Einheit weniger ärztliches Personal (3–4 Personen) als beim „klassischen“ UaP (5–6 Personen) erforderlich war, wurden jeweils mindestens 2–3 wissenschaftliche und technische Mitarbeiterinnen und Mitarbeiter eingesetzt, welche nun wiederum an anderer Stelle kompensiert werden mussten. Zudem lässt sich der personelle und zeitliche Aufwand, welcher in der relativ kurzen Vorbereitungsphase zur Adaptation des UaP und Konzeption des videobasierten Formats erforderlich war, schwer quantifizieren.

Als Limitation dieser Studie ist die geringe Rücklaufquote von 16,8 % zu betrachten, welche die Aussagefähigkeit und Verallgemeinerbarkeit sicher einschränkt. Dieser Aspekt sollte bei Folgestudien berücksichtigt werden mit dem Ziel, die Rücklaufquote durch z. B. geeignete Anreizsysteme zu erhöhen. Ein Faktor ist hier sicher der zeitliche Abstand von mehreren Monaten zwischen letzter Unterrichtseinheit und dem Versenden des Fragebogens. An der medizinischen Fakultät der Autoren ist der Erhalt eines „Scheins“ zudem nicht an eine Abgabe der Evaluation gebunden, was sicherlich ein geeignetes Mittel wäre, eine hohe Rücklaufquote zu gewährleisten.

Auch wenn bei der Übertragung in den Hörsaal die Hygienevorschriften gemäß der entsprechenden Eindämmungsverordnung [7] streng eingehalten wurden, muss prinzipiell hinterfragt werden, warum zu Pandemiezeiten, in einer Phase ohne Impfstoff, die Videoübertragung nicht z. B. als Streaming zu den Studierenden nach Hause erfolgte. Aufgrund des Präsenzcharakters der Veranstaltung war es notwendig, die konkreten Teilnehmer zu identifizieren und authentifizieren, was durch persönliche Anwesenheit im Hörsaal gut möglich war, bei einer Videoübertragung nach außen zum damaligen Zeitpunkt jedoch technisch kaum umsetzbar gewesen wäre. Zudem bestanden datenschutzrechtliche Bedenken: So wäre es nicht zweifelsfrei zu überprüfen gewesen, ob ein nicht legitimierter Personenkreis ebenfalls zusehen würde oder eine Videoaufzeichnung auf dem heimischen Rechner erfolgen würde.

Die Arbeit an dieser Studie legte noch einen weiteren grundlegenden Aspekt offen: So herrscht allgemein kein Konsens, was die Terminologie für die verschiedenen Formen der medizinischen Lehre betrifft. So werden beispielweise Begriffe wie „virtuell“ oder „online“ nicht immer semantisch korrekt und nicht einheitlich eingesetzt bzw. können missverstanden werden. Lakner et al. unterscheiden zwischen „virtueller Lehre“ (einmal erstellte, permanent abrufbare Lehrangebote/Streaming ohne Echtzeitpräsenz des Lehrenden) und „Teleteaching“ (präsenzähnliche, virtuelle Kommunikation räumlich verteilter Lernender und Lehrender, die auf verbale Äußerungen ebenso zurückgreifen kann wie auf Gestik und Mimik und die Echtzeitpräsenz des Lehrenden erfordert [Live-Ereignis]) jeweils mit oder ohne Interaktionsmöglichkeit des Studenten (M. Neudert, Dresden, persönliche Kommunikation und [6]). Das hier vorgestellte Lehrkonzept wird nach Auffassung der Autoren am ehesten mit einem „interaktiven, videobasierten Distanzunterricht am Patienten“ beschrieben oder auch „Teleteaching mit Interaktion“.

In den Fragen an die Studierenden wurde noch die Begrifflichkeit eines online-basierten interaktiven UaP-Lehrkonzepts verwendet, da sich die Problematik der Terminologie erst bei der Erstellung des Manuskripts herausdifferenziert hat und daraufhin diskutiert wurde. Die korrekte inhaltliche Zuordnung dieser Beschreibung war für die Studierenden jedoch zweifelsfrei möglich, da sie ja an der Veranstaltung teilgenommen hatten.

Während der Unterricht am Patienten seit der Zeit von Sir William Osler im späten 19. Jahrhundert eine bedeutende Rolle in der Medizinerausbildung einnimmt [14], wird diesem Lehrkonzept weltweit in den letzten Jahren immer weniger Platz eingeräumt, sei es durch zunehmende Verlagerung der Diagnosefindung von klinischen Befunden auf apparative und laborchemisch gestützte Methoden, sei es durch strukturelle Veränderung der lehrenden Krankenhäuser mit einer deutlichen Erhöhung des Patientendurchsatzes, was zum einen die ärztliche Arbeitsbelastung erhöht, wie auch die Patientenverfügbarkeit für die Lehre senkt [11]. Als weitere Gründe werden zeitliche Faktoren oder auch fehlende praktische und theoretische Fähigkeiten der Studierenden genannt [11]. Eine zukünftige Implementierung von Aspekten des Distanz-UaP wurde in den offenen Antworten mehrfach vorgeschlagen und könnte den klassischen UaP sinnvoll ergänzen. Miller [8] sieht in der pandemiebedingten Anwendung von Videoübertragungen beim Bedside-Teaching sogar die Chance auf eine Rückbesinnung auf wichtige Aspekte dieses Lehrkonzepts, welche beim klassischen UaP im klinischen Alltag zunehmend in den Hintergrund geraten sind. So kann hier wieder strukturierte Konsensfindung im Team unter dauerhafter professioneller Anleitung und Aufsicht gewährleistet werden, was beim klassischen UaP im realen klinischen Alltag nicht immer gut möglich ist.

## Fazit für die Praxis

- Interaktiver, videobasierter Distanzunterricht am Patienten (Teleteaching mit Interaktion) kann den direkten Patientenkontakt nicht ersetzten, stellt jedoch speziell im HNO-Gebiet eine gute Alternative dar, wenn durch pandemiebedingte Umstände ein Präsenz-UaP mit direktem Patientenkontakt nicht möglich ist.
- Einzelne Aspekte, wie beispielsweise die Bildübertragung der klinischen Befunde oder die Konsensfindung im Team, könnten auch zukünftig den klassischen UaP ergänzen.
